# Microorganisms and Their Metabolic Capabilities in the Context of the Biogeochemical Nitrogen Cycle at Extreme Environments

**DOI:** 10.3390/ijms21124228

**Published:** 2020-06-13

**Authors:** Rosa María Martínez-Espinosa

**Affiliations:** 1 Biochemistry and Molecular Biology Division, Agrochemistry and Biochemistry Department, Faculty of Sciences, University of Alicante, Ap. 99, E-03080 Alicante, Spain; rosa.martinez@ua.es; Tel.: +34-965903400 (ext. 1258); 2 Multidisciplinary Institute for Environmental Studies “Ramón Margalef”, University of Alicante, Ap. 99, E-03080 Alicante, Spain

**Keywords:** ammonium oxidation, archaea, biogeochemistry, denitrification, nitrogen assimilation, nitrogen cycle

## Abstract

Extreme microorganisms (extremophile) are organisms that inhabit environments characterized by inhospitable parameters for most live beings (extreme temperatures and pH values, high or low ionic strength, pressure, or scarcity of nutrients). To grow optimally under these conditions, extremophiles have evolved molecular adaptations affecting their physiology, metabolism, cell signaling, etc. Due to their peculiarities in terms of physiology and metabolism, they have become good models for (i) understanding the limits of life on Earth, (ii) exploring the possible existence of extraterrestrial life (Astrobiology), or (iii) to look for potential applications in biotechnology. Recent research has revealed that extremophilic microbes play key roles in all biogeochemical cycles on Earth. Nitrogen cycle (N-cycle) is one of the most important biogeochemical cycles in nature; thanks to it, nitrogen is converted into multiple chemical forms, which circulate among atmospheric, terrestrial and aquatic ecosystems. This review summarizes recent knowledge on the role of extreme microorganisms in the N-cycle in extremophilic ecosystems, with special emphasis on members of the Archaea domain. Potential implications of these microbes in global warming and nitrogen balance, as well as their biotechnological applications are also discussed.

## 1. Introduction

Extreme microorganisms, also called “extremophiles” (from Latin *extremus* meaning “extreme” and Greek *philiā* (φιλία) meaning “love”) are organisms that thrive in physically or geochemically extreme conditions that are usually detrimental to most life on Earth [1,2]. Thus, these microorganisms inhabit environments characterized by significantly extreme temperatures and/or pH values, high or low ionic strength, high pressure, or scarce nutrients availability. To grow under optimal conditions in these extreme ecosystems, they have evolved molecular adaptations affecting physiology, metabolism, cell signaling, etc. [3,4,5,6,7]. It was considered that the extremophile organisms were sparse, and their presence limited to extreme ecosystems. However, studies conducted over the past two decades have shown that they are more varied and abundant than initially thought [8,9]. Extremophilic organisms are widespread within the three domains of life, but most of them are prokaryotic organisms belonging to the archaea domain [10]. The extremophilic organisms can be classified following different criteria, but the most useful classification establishes groups based on the environmental conditions in which they grow optimally (see Section 2). Due to the extreme patterns characterizing their metabolism and physiology, extremophiles have become good models of study in the following fields of knowledge:

(i) Understanding the limits of life on Earth. The study of extremophiles improves understanding of the physicochemical parameters defining life on Earth and may provide insight into how life on Earth originated [11,12,13,14]. The postulations supporting that primitive Earth had extreme environmental conditions and that life arose in hot environments have led to the theory that extremophiles could be vestiges of primordial life forms, and thus are models of ancient life [15,16,17,18]. Thus, the improvement in the knowledge about primitive life conditions and extremophiles could span the study of their biology and of the global biogeochemical cycling of elements, particularly on N-cycle, which is the aim in this review (Section 3) [19,20]. The number of studies about extremophiles in connection with N-cycle is still scarce, but some of them have analyzed global distribution and diversity of extremophiles related to nitrogen availability as well as biochemical characterization of some reactions of N-cycle driven by extremophiles [19,21,22].

(ii) Exploring the possible existence of extraterrestrial life (Astrobiology). Extremophiles, especially those thriving under multiple extremes, are good model organisms to carry out research in multiple disciplines, spanning areas such as the study of adaptations to harsh conditions, to the biogeochemical cycling of elements. Thus, extremophile research has implications for origin of life studies and the search for life on other planetary and celestial bodies [23,24,25,26,27,28]. Extremophiles inhabiting cold environments are of interest in this field, as most of the bodies in the solar system are frozen. On the other hand, some extremophilic microbes show unusual biochemical properties, which are also of interest to Astrobiology since extraterrestrial environments may favor life-forms that use or are built from elements not typically found in life on Earth [18,29,30,31]. Thus, insights on the knowledge of biogeochemical cycles (C, N, S, and O, among others) in extreme environments could be used as a model to explore the cycling of elements in Astrobiology or to look for astrobiological signatures worldwide [32,33,34].

(iii) Potential applications in biotechnology. The molecular adaptations of extremophiles to their environments make them a powerful natural source of molecules and even metabolic pathways to explore. This is an important field for research and industrial production of marketed biomolecules as carotenoids (pigments), antibiotics, biodegradable plastics, such as polyhydroxyalkanoates (PHA), or enzymes. Extreme enzymes, for instance, are useful in industrial procedures due to their ability to remain active under the severe conditions typically employed in these processes [35,36,37,38]. In the case of biotechnological applications associated with N-cycle, it is worth mentioning the potential uses of denitrifying extremophilic microbes for bioremediation approaches or as a source of enzymes for the design of electrons to measure nitrate/nitrite, as it is discussed in detail in Section 4 [39,40].

In summary, microorganisms are essential for the reactions of biogeochemical cycles, and extremophilic microbes are not an exception [41,42,43]. Among the crucial cycles for life on Earth, nitrogen and carbon cycles are currently facing an unprecedented set of comprehensive anthropogenic changes, mainly due to fossil combustion, agricultural practices, and industry [44,45]. The nitrogen cycle (N-cycle) is one of the most important biogeochemical cycles in nature, because thanks to it, nitrogen is converted into multiple chemical forms, which circulate among the atmosphere, and terrestrial and aquatic ecosystems. Nitrogen is part of the main building blocks of life (including DNA, RNA, and proteins) and it is stored in all of the Earth’s geological reservoirs, including the crust, the mantle, and the core [46]. Besides, N_2_ is the dominant gas in the Earth’s atmosphere and it is indispensable for sustaining human activities through its role in the production of food, animal feed, and synthetic chemicals. This has encouraged significant anthropogenic mobilization of reactive nitrogen and its emissions into the environment resulting in severe disruption of the nitrogen cycle [47,48].

The contribution of microorganisms at a global scale in the interchange between nitrogen forms is relevant and considering that extreme habitats are mostly occupied by extremophilic microbes, it is possible to assume that they could play relevant roles catalyzing reactions of the N-cycle in several extreme ecosystems, such as salty environments, soda lakes, mine sites, hot springs, volcano, etc. [19,49,50,51]. The global area occupied by extreme ecosystems is not precisely estimated, but it is known to be a significant extension (glaciers, volcanoes, desert, arid, and semiarid regions, etc.). In addition, various anthropogenic activities are changing the environment alarmingly, contributing to pollution, and increasing the occupation of these extreme ecosystems. Consequently, the role of extreme microbes may be even more significant in the near future as these extreme ecosystems increase in extension and prevalence. Therefore, it is necessary to draw the attention of the scientific community to focus efforts on the development of more research, at both basic and applied levels, which will allow a better understanding of microbial populations in extreme ecosystems and their role in the development of biogeochemical cycles. This review summarizes recent knowledge on the role of extreme microorganisms in the biogeochemical N-cycle, with special emphasis on members of the archaea domain (which constitute the major populations in most of these environments). Finally, some of the main applications of N-cycle reactions carried out by extremophilic archaea are discussed.

## 2. Classification of Extreme Microorganisms

Extremophiles can be divided into two broad categories: extremophilic organisms, which require one or more extreme conditions to grow, and extremotolerant organisms, which can tolerate extreme values of one or more physicochemical parameters though growing optimally at “normal” conditions [1]. In contrast, the term “mesophile” refers to microbes growing best in moderate temperature (typically between 20 and 45 °C (68 and 113 °F)) and usually at pHs between 6 and 8 [52].

Extremophilic microorganisms are mainly classified according to the conditions in which they grow. Table 1 displays the main groups established following this criterion. Some extremophiles are adapted simultaneously to multiple stresses, and they are called “Polyextremophiles”. This is the case of haloalkalophiles (the combination of halophilic and alkalophilic profiles: salt concentration between 2 and 4 M and pH values of 9 or above) or thermoacidophiles (the combination of thermophilic and acidophilic profiles: temperatures of 70–80 °C and pHs between 2 and 3) [53,54,55,56].

Apart from these groups of extremophilic microbes, some microbes growing under mesophilic conditions, pH values around neutrality (6.5–8) and moderate ionic strength, show unusual metabolic capacities able to tolerate or even metabolize significant concentrations of heavy metals or other compounds with toxic effects for most of the organisms. This is the case of microbes tolerating or metabolizing arsenic [57,58,59], cadmium [60,61], zinc [62,63], or mercury [64,65], among other toxic elements. However, these microorganisms cannot be considered extremophiles based on classical definitions.

Although extremophiles include members of all three domains of life, i.e., bacteria, archaea, and eukarya, most of them belong to archaea. Thus, some archaea are among the most hyperthermophilic, acidophilic, alkaliphilic, and halophilic microorganisms known up to now. Some good examples of these hyper extreme phenotypes are the following: the archaeon *Methanopyrus kandleri* strain 116 grows at high temperatures above 98 °C and up to 122 °C (252 °F, the highest recorded temperature) [66], while the genus *Picrophilus* (e.g., *Picrophilus torridus*) includes the most acidophilic organisms currently known, with the ability to grow at a pH of 0.06 [67]. On the other hand, good examples of extremely halophilic microbes are found within the families *Halobacteriaceae* and *Haloferacaceae* [68]. Within the bacteria domain, it is worth mentioning not only cyanobacteria but also genus, such as *Thermus* (from which several enzymes with potential uses in biotechnology have been isolated) or *Salinibacter*, which have representatives inhabiting extremely hot or salty environments, respectively [69,70,71]. Among eukaryotes, several genera of fungi (alone or in symbiosis) have been isolated from extreme environments, such as mining regions, alkaline ecosystems, hot or cold deserts, the deep ocean, and in hypersaline regions, such as the Dead Sea [72,73,74,75]. Nevertheless, in terms of high resistance to extreme conditions, one of the most impressive eukaryotic polyextremophiles is the tardigrade, a microscopic invertebrate able to go into a hibernation mode, thus surviving at temperatures from −272 °C to 151 °C, vacuum conditions (imposing extreme dehydration), pressure of 6000 atm, as well as exposure to X-rays and gamma-rays [76,77]. 

## 3. Extreme Microorganisms in the Context of Biogeochemical Nitrogen Cycle

### 3.1. General Overview of the Role of Microorganisms in N-Cycle

**Figure 1:**
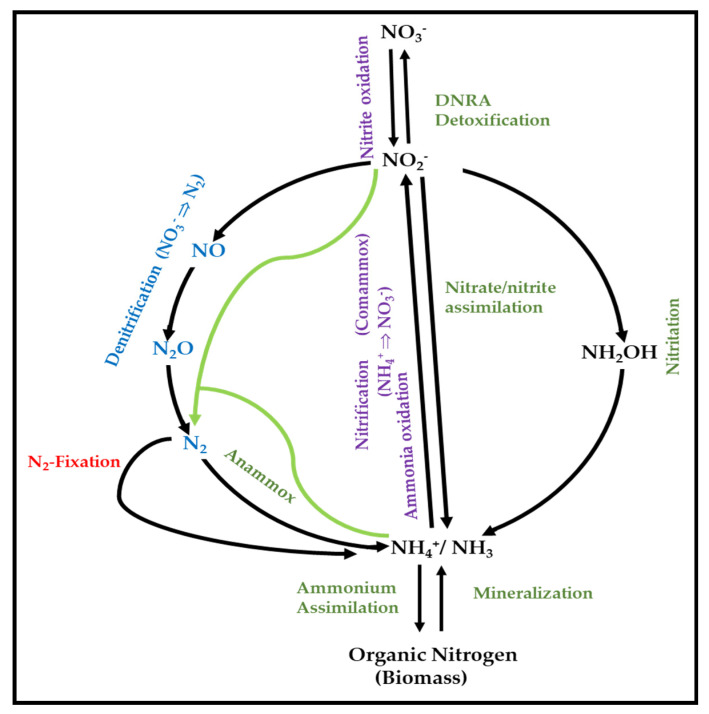
Major processes of the N-cycle (adapted from Martínez-Espinosa, 2019) [99].

The N-cycle is one of the most important biogeochemical cycles of the Earth, with large natural flows of nitrogen from the atmosphere into terrestrial and marine ecosystems through several biological processes [78,79]. It involves pathways such as nitrogen fixation, nitrification, nitrate/ammonium assimilation, dissimilatory nitrate reduction to ammonia (DNRA), anaerobic ammonia oxidation (ANAMMOX), complete ammonia oxidation (COMAMMOX), and denitrification (Figure 1). In brief, NO_3_^−^ and NH_4_^+^ can be used as nitrogen sources for growth under aerobic conditions (assimilatory nitrate reduction/ammonium assimilation). NO_3_^−^ can also be the final electron acceptor for respiration under anoxia (denitrification) or an electron acceptor in a redox process aiming at the removal of excess of reductant power through dissimilatory nitrate reduction. 

Dissimilatory NO_3_^−^ reduction, NO_3_^−^ respiration, or denitrification are often used equivalently in the literature, and the intermediate products are NO_2_^−^, NO, and N_2_O [78,79]. However, the dissimilatory pathway refers to non-assimilatory reactions that are not directly coupled to the generation of proton motive force. Dissimilatory nitrate reduction to ammonium (DNRA) is also possible under anaerobic or microaerobic conditions. On the other hand, NO_2_^−^ could be reduced to NH_4_^+^, which is then excreted, thanks to the process called NO_3_^−^/NO_2_^−^ ammonification. Some organisms can oxidize either NH_4_^+^ or NO_2_^−^ by using a pathway called nitrification, while other organisms, such as some planctomycetes, oxidize NH_4_^+^ and utilize NO_2_^−^ as a respiratory electron acceptor in a pathway named ANAMMOX [80,81,82]. Recently, the discovery of new members of the *Nitrospira* genus, able to catalyze both nitrification steps (ammonia oxidation and nitrite oxidation), has allowed the proposal of ‘COMAMMOX’ organisms, also called “complete ammonia oxidizers” [83,84,85]. 

Finally, (di)nitrogen fixation allows several microorganisms to reduce N_2_ to NH_4_^+^ to supply nitrogen to plants. Plants are not able to fix their own nitrogen, but a few of them (mainly legumes) fix nitrogen via symbiotic anaerobic microorganisms (mainly rhizobia). Thus, nitrogen fixation, along with photosynthesis is the basis of all life on Earth [86,87,88]. Free-living diazotrophic microorganisms also play an important role in carrying out nitrogen fixation in ecosystems such as oceans [89] (which cannot be considered extremophilic environments) and extreme environments such as glacier fore field environments [90], desert-like ecosystems [91], or hot springs [92]. Thanks to these microbes, nitrogen fixation is revealed as a crucial pathway for building labile nitrogen stocks and facilitating higher plant colonization in oligotrophic glacier fore field soils [90,93] or hot springs [92,94,95,96]. Chemolithotrophic nitrogen fixation at high temperatures (up to 92 °C) has attracted scientists researching the early evolution of life and the nitrogen cycle, and deep-sea hyperthermophilic methanogens and their nitrogen fixation processes have been extensively examined [96,97,98].

Most of the mentioned pathways are thriven by prokaryotes, although nitrogen fixation also involves plants and their associated rhizobia and nitrate/ammonium assimilation can be observed in both prokaryotes and eukaryotes.

### 3.2. Metabolic Pathways of N-Cycle Carried out by Extremophiles

Regarding extremophilic microbes, recent research has revealed that not only bacteria but also archaea may contribute to several pathways of the N-cycle in extreme environments. The results already reported are scarce compared to non-extreme ecosystems. However, the current evidences from molecular ecology, genomics, metagenomics, biochemical, and physiological studies mainly offer details about their potential role in the following N-cycle pathways: (i) aerobic or (ii) anaerobic ammonium oxidation, (iii) anaerobic denitrification, and (iv) nitrate/ammonium assimilation. The main findings related to these pathways are summarized following:

(i) Aerobic ammonium oxidation is the process of converting ammonium to nitrate and thus links the regeneration of organic nitrogen to fixed nitrogen loss by denitrification. Ammonium oxidation is critical to global nitrogen cycling and is often thought to be driven only by ammonia-oxidizing bacteria phylogenetically included in the group of Proteobacteria (ammonia-oxidizing bacteria, AOB), that are autotrophic and obligatory aerobic [100,101]. At the beginning of this century, the finding of new ammonia-oxidizing organisms belonging to the archaeal domain challenges this perception. Recent studies have stated that ammonia-oxidizing archaea (AOA) can be both abundant and diverse in aquatic and terrestrial ecosystems studies, and at least some AOA have a high substrate affinity for ammonia being able to grow under extremely oligotrophic conditions [102,103]. However, the global characterization of this pathway in extremophilic environments is still scarce, and most of the results reported come from studies done with members of Crenarchaea and Thaumarchaea (archaea domain). Some of these works have been conducted in natural environments, such as soils or the deep-subsurface of hydrothermal aquifers, in which thermophilic archaea belonging to Thaumarchaeota sustain this process [104]. Regarding halophilic environments, aerobic ammonium oxidation has been reported from oceanic ecosystems, such as coastal marine wetlands, anoxic oceanic depth zones, or coral reefs [105,106,107]. Consequently, two major microbial groups are now believed to be involved in ammonia oxidation: chemolithotrophic ammonia-oxidizing bacteria (AOB) and ammonia-oxidizing archaea (AOA) [103,108]. 

(ii) Anaerobic ammonium oxidation (ANAMMOX) is the process of oxidizing ammonium through the reduction of nitrite. This pathway was first described in a denitrifying pilot plant reactor [109], and the enzymes involved in this process have been described in detail for several Anammox bacteria [80,110]. Wastewater has been a good source for the isolation of new species showing ANAMMOX capacity. However, microbes carrying out ANAMMOX processes have also been isolated from natural environments, and many of them are mesophilic bacteria. Extremophilic microbes able to carry out the ANAMMOX process have been recently described, too, not only in pilot plants, brines, or sludges but also in natural ecosystems, such as some freshwater extreme environments, hot springs, and deep-sea hydrothermal vents [111,112,113]. ANAMMOX within the domain archaea has been recognized as a critically important process in the environment, and particularly in the ocean, but from an accurate point of view based on definitions, oceans cannot be considered extreme environments [114]. Thus, the degree of characterization of this pathway in extremophiles is relatively low compared to the knowledge on ANAMMOX in mesophilic bacteria and, consequently, more research is needed on this topic in the future. 

(iii) Anaerobic denitrification is an anaerobic respiratory pathway in which nitrate is reduced to dinitrogen. Some denitrifiers are complete, i.e., nitrate is fully reduced to dinitrogen thanks to four key enzymes: respiratory nitrate reductase (Nar), respiratory nitrite reductase (Nir), nitric oxide reductase (Nor), and nitrous oxide reductase (Nos). However, the process is often incomplete (partial denitrification), leading to the release of the gaseous intermediates NO and N_2_O, which affect the environment [39,115]. Denitrification has been extensively described so far, but regarding extremophilic microorganisms, most of the studies have been reported from both thermophilic bacteria and archaea and halophilic archaea. In the case of thermophilic bacteria, denitrification has been studied in detail in a few species of the genera *Thermus* [116,117,118]. Related to extreme archaea, denitrification has been described in several species of halophilic archaea [39,119,120,121] as well as in the thermophilic archaeon *Pyrobaculum aerophilum* [122,123,124]. A few examples of anaerobic denitrification have also been reported from moderately halophilic bacteria [125,126,127]. The enzymes catalyzing anaerobic denitrification in extremophilic archaea and bacteria have been characterized from a biochemical point of view (mainly respiratory nitrate and nitrite reductases) and share some structural features [39,128,129]. However, more effort must be made in the future to elucidate the mechanisms regulating denitrification in other extremophiles, such as halophiles (bacteria or archaea).

(iv) Finally, nitrate and ammonium assimilation have been very well characterized in symbiotic microbes, mesophilic bacteria, algae, plants, and fungi. However, the knowledge about these processes in extreme environments is still scarce and mainly limited to a few members of the *Haloferacaceae* family (halophilic archaea) or a few thermophiles (bacteria). In the case of haloarchaea, nitrate assimilation involves two enzymes: assimilatory nitrate reductases (Nas) and assimilatory nitrite reductases (Nir). [130,131,132,133]. The ammonium produced from nitrate/nitrite reduction or ammonium directly taken up from the environment can be assimilated through the glutamine synthetase/glutamate synthase cycle (GS/GOGAT) (when the intracellular concentration of ammonium is relatively low) [134,135] or thanks to glutamate dehydrogenase (GDH) (if the cytoplasmic ammonium concentration is significantly high) [136]. Thus, nitrate/ammonium assimilation in halophilic archaea is similar to the processes described from cyanobacteria and bacteria. Regarding thermophiles, most of the studies reported at the time of writing this review are focused on ammonium assimilation. This process has been partially characterized from a biochemical point of view in thermophilic bacteria, such as *Thermus thermophilus*, in which GDH activity was reported two decades ago [137,138] or *Bacillus caldolyticus*, from which two GSs have been isolated and characterized [139]. GSs enzymatic activity, or ammonium assimilation capacity in general, has also been predicted on the base of genomic analysis from the thermophilic bacterium, *Thermotoga maritima* [140], or from the acidic and chemolithoautrophic bacterium *Leptospirillum ferriphilum* ML-04 [141].

## 4. Potential Applications of N-Cycle Pathways Driven by Extremophiles in Biotechnology and in Studies on Climate Change and Environmental Global Warming

### 4.1. Wastewater Treatments and Bioremediation

Wastewater treatments (WWT), such as the breakdown of sewage influent, are generally performed by microorganisms and biological nitrogen removal (BNR) is a critical process in the treatment. Recently, there have been new microbial communities discovered capable of performing BNR with novel metabolic pathways [142]. Besides, extremophilic microbes dealing with different nitrogen compounds at high concentrations have also been characterized [143]. Usually, these microbes have advantages over canonical ammonium oxidizers, nitrifiers, or denitrifiers, such as higher substrate affinities, better physicochemical tolerances, and/or less greenhouse gas emission. It is important to highlight that nitrification and aerobic ammonium oxidation driven by extremophilic microbes belonging to archaea have been recently described, and they could be promising metabolic pathways for wastewater or sludge treatments in combination with denitrification (both bacteria or archaea) [98,144,145,146,147,148]. Regarding denitrification, it has been demonstrated that some haloarchaeal species, such as *Haloferax mediterranei,* are able to metabolize high nitrate and nitrite concentrations under aerobic, microaerobic, and anaerobic conditions (some of the species efficiently remove up to 2 M NO_3_^−^ and up to 60 mM NO_2_^−^, which are the highest concentrations currently described). Consequently, these species have been proposed as good model organisms to design new strategies for the removal of nitrogen in wastewater treatments or the treatments of brines [39,115,143]. Thus, biological approaches based on complete denitrifiers take advantage of specific groups of microorganisms involved in nitrogen cycling to remove reactive nitrogen from reactor systems by converting nitrate, nitrite, or ammonia to nitrogen gas [146]. Recent studies have shown that thanks to the denitrification route, and particularly thanks to the enzyme respiratory nitrate reductase, certain extreme denitrifying microorganisms (archaea and bacteria) can reduce perchlorate to chlorate, and chlorate to chlorite [149]. Thus, not only nitrate/nitrite but also perchlorate and chlorate can be removed from wastewater and brines by the reaction catalyzed by the respiratory nitrate reductase (this is the first enzyme involved in denitrification, and it is able to recognize nitrate, perchlorate, and chlorate as substrates) [150]. Over the last decade, perchlorate (ClO_4_^−^) and chlorate (ClO_3_^−^) have been detected in water supplies, groundwaters, agricultural crops, and even in soils as a result of human activities [151]. On the one hand, perchlorate is used in the manufacture of propellants, explosives, and pyrotechnic devices [152]. The main concerns about perchlorate toxicity are its interference with iodide uptake by the thyroid gland, and the related potential carcinogenic effects [153]. On the other hand, chlorate is present in several herbicides and defoliants, and it is released when chlorine dioxide (ClO_2_^−^) is used as a bleaching agent in the paper and pulp industry [154]. Thus, denitrification carried out by these microbes could sustain the removal of other toxic compounds, such as (per)chlorates, apart from nitrogenous compounds to treat urban or industrial wastewaters [150,155].

Finally, bioremediation-based processes use microorganisms for the degradation or removal of contaminants (bioaugmentation, biodegradation, bioleaching, etc.). Pollution of soils, sediments, and groundwater is a matter of concern at the global level; thus, pollutant removal has become a priority worldwide. Currently, bioremediation has emerged as an effective solution for these problems, and, indeed, the use of extremophilic microorganisms in bioremediation has been tested successfully [40]. Most bioremediation research has focused on processes performed by members of the domain bacteria; however, archaea are well suited for bioremediation in extreme conditions, such as halophilic or acidophilic environments. In other conditions, archaea collaboratively work alongside bacteria during biodegradation [156]. Although most of the bioremediation processes involving extremophilic microorganisms include halophilic hydrocarbon degradation, acidophilic hydrocarbon degradation, hydrocarbon degradation, or dehalogenation, it is possible to assume their potential use in the removal of nitrogenous compounds from soils thanks to denitrification and/or aerobic ammonium oxidation [39,40].

### 4.2. Environmental Studies

Climate change, environmental global warming, and anthropogenic nitrogen deposition are three of the main current concerns worldwide [44,157]. Evaluating their cumulative effects provides a more holistic view of ecosystem vulnerability to human activities, which would better inform policy decisions aimed to protect the ecosystems. Changes of global climate modify key processes in terrestrial and freshwater ecosystems related to nitrogen cycling and availability as well as the response of ecosystems to nitrogen addition in terms of carbon cycling, acidification, and biodiversity [157]. Therefore, the knowledge of N-cycle and microbial activities must improve for better understanding the magnitude of climate effects on ecosystem response to N.

A deep revision done at the time of writing this work shows that although N-cycle has been extensively studied worldwide, including extremophilic environments (mainly thermophilic (hot springs) or halophilic types (oceanic ecosystems, salted ponds or marshes)), it has not been explored yet in extreme environments, such as volcano surroundings, drylands, psychrophilic or barophilic environments. Recent results from metagenomics, proteomics, massive analysis of environmental genomes, etc., suggest that the role of extremophilic microbes in nitrogen cycle is more relevant than thought so far [44,158]. Among extremophilic ammonia oxidizers, members of archaea show the most extreme phenotypes. The widespread occurrence and diversity of ammonia-oxidizing archaea suggest their contribution to the nitrogen cycle is of global significance and higher than initially thought [147,159]. Their distribution appeared limited to low- and moderate-temperature environments until the recent finding of a diagnostic membrane lipid, crenarchaeol, in terrestrial hot springs. These findings greatly extend the upper-temperature limit of nitrification and document that the capacity for ammonia oxidation is broadly distributed among the Crenarchaeota [147,160].

In halophilic ecosystems, such as salt marshes or salted ponds, the low oxygen solubility and high ionic strength promotes the development of denitrification in those geographical regions in which nitrate or nitrite are present in soil or water (from natural sources or as part of the pesticides and fertilizers used for agricultural purposes) [39,44,105,161]. In those environments, partial denitrification results in the emission of NO and N_2_O gases to the atmosphere, thus contributing to global warming [39,51,105]. Studies on nitrogenous gas emissions by haloarchaea at a laboratory scale have demonstrated that these emissions are not negligible [115]. Therefore, it would be interesting to quantify the magnitude of NO and N_2_O emissions from arid soils and brines in which these halophilic denitrifying microbes constitute predominant microbial populations [115].

One of the best-analyzed phenomena regarding connections between N-cycle, global warming, and other environmental changes is the effect of the increased use of nitrogenous fertilizers in agriculture worldwide. The abusive use of fertilizers and pesticides has significantly altered the global N-cycle because of the release of nitrogenous gases due to the metabolic activities of soil microbes. As mentioned, the emission of nitrous oxide (N_2_O) contributes to the global greenhouse gas accumulation and the stratospheric ozone depletion [47,162,163]. These connections have been explored in detail mainly in forest ecosystems [164], pasture soils [165,166], and standard agricultural soils or agricultural soils suffering dramatical pH changes [164,165,166,167,168,169,170]. As a method to reduce the emission of nitrogenous gasses, complete denitrification appears as a potential strategy to reduce N_2_O emissions by enhancing the activity of N_2_O reductase (NOS) in the denitrifying microbial community [171]. Consequently, denitrifying microbial communities could act as sources or sinks for nitrogenous gases [172]. However, studies on this topic from extreme environments are yet to come. 

### 4.3. N-Cycle Enzymes

Microbial communities constituting the main populations in extreme environments have become a focus of scientific interest owing to the unique properties of the biocatalysts they produce (extremozymes). Extremozymes can cope with industrial process conditions (high temperatures, high salt concentrations, low water availability, etc.) due to their extreme stability under the mentioned parameters. For this reason, extremozymes are in demand for large-scale production in several chemical industries, biotransformation, and in the field of bioremediation [173]. In that context, extremophilic bacteria, fungi, and archaea are a valuable source of novel enzymes for biotechnology. Thus, thermophilic proteins, piezophilic proteins, acidophilic proteins, and halophilic proteins have been studied during the last few years. Among them, amylases, proteases, lipases, pullulanases, cellulases, chitinases, xylanases, pectinases, isomerases, esterases, and dehydrogenases have great potential application for biotechnology, such as in agricultural, chemical, biomedical, and biotechnological processes [173,174,175,176]. 

In the context of this review, enzymes and accessory proteins (such as electron transfer proteins) involved in denitrification have yielded the best applications. Those enzymes have been used to prepare enzymatic cocktails and biosensors. Due to the importance of nitrates and nitrites (NO_x_^−^) as contaminants in soils and waters, two main lines of biosensors have been investigated: (i) biosensors based on the use of whole cells (the biosensor detects products of cellular nitrogen metabolism), and (ii) systems based on the immobilization of denitrification enzymes on a matrix. In the first type of biosensors, the cells are placed in a reaction chamber in which the reduction of NO_3_^−^ to N_2_O occurs. The reaction is usually measured by a specific nitrous oxide microelectrode [177,178,179]. In the second generation of biosensors, modified cell biosensors were constructed by fusing a reporter gene to a promoter element that is induced by the presence of a target compound (nitrate or nitrite). Thus, a whole-cell fluorescence biosensor based on recombinant *Escherichia coli* allowed the determination of nitrate without the interference of phosphate, chloride, or nitrite [180]. In the second type of biosensors, the enzymes (mainly nitrate and nitrite reductases isolated from mesophilic denitrifying bacteria, such as *Paracoccus*, *Alcaligenes,* or *Desulfovibrio* species) are immobilized on different materials to monitor the concentration of NO_x_^−^. The immobilization of the enzymes improves the stability and half-life of the enzymes, making the system robust and sensible. Other aspects, such as quick response, high selectivity, and sensitivity, low cost, and portable dimensions, are also inherent to electrochemical biosensors based on redox enzymes involved in N-cycle [181,182,183]. Apart from the enzymes catalyzing the four main reactions of denitrification, other accessory proteins involved in denitrification, such as cytochrome c, have also been tested as part of a biosensor to monitor nitrate, hydrogen peroxide or superoxide [184].

One of the major problems that these biosensors have is that the whole cells or the isolate enzymes should work under specific conditions that preserve high stability and enzymatic activity. These conditions usually are room temperature (or temperatures between 15–30 °C), neutral pH values, low ionic strength, etc. Consequently, those biosensors are not useful to quantify nitrate and nitrite in the field when working with environmental samples, such as brines, acid or basic wastewaters, salted soils, etc. In that context, extremophilic denitrifiers are good candidates to make innovative biosensors. At the time of writing this work, there are only a few studies focused on the use of a psychotropic bacteria-based NO_x_^−^ biosensor to analyze marine sediments. This biosensor can be used at low temperatures (<2.5 °C) and high salinity (around 35%) [185]. On the other hand, several studies about N-cycle in haloarchaea suggest that some denitrifying species and their isolated enzymes are highly efficient, catalyzing the reduction of NO_x_^−^ under both aerobic and anaerobic conditions [39,51,115,128,143]. Consequently, new biosensors could be developed using whole haloarchaeal cells or even respiratory halophilic nitrate and nitrite reductases.

## 5. Conclusions

Biochemical cycles, as well as microbial diversity in extreme environments, are still poorly described. Considering that N-cycle pathways are mainly driven by microorganisms, more efforts must be made to understand their physiology and metabolism as well as their ecological relevance to modulate N:P:K balances and the interconversion of nitrogenous compounds in extreme ecosystems. Besides, although it is widely assumed that these microorganisms could be of high interest in terms of biotechnological applications due to N-cycle pathways, only a few studies at laboratory scale have been carried out. Taking all the previous aspects into account, several questions arise: (i) How significant is the contribution of extremophilic microbes in the global biogeochemical N-cycle, in climate change, and greenhouse gas emissions? (ii) Are extremophilic microbes involved in N-cycle good candidates for biotechnological applications at large scale (wastewater/sludge treatments, etc.)?

The design and development of research to address these questions is quite a challenge for the next decade. It is relevant to emphasize that natural events, as well as anthropogenic activities, are contributing to global warming and climate change. Consequently, physicochemical parameters directly connected to N-cycle have been significantly affected, especially during the last two decades. Besides, the size and prevalence of arid and semiarid regions, among other types of extreme environments, are increasing [44,115]. In this context, understanding biogeochemical cycles in extreme environments is of great soundness and one of the aims to overcome soon. Policies on global warming and climate change must be revised and implemented to address the main consensus on natural resources or emissions of greenhouse gases. Particularly, policies to avoid environmental degradation and to mitigate N_2_O emissions from natural or artificial biological nitrogen removal systems must be designed and implemented [186,187].

## Figures and Tables

**Table 1:** General classification of extremophilic microorganisms (adapted from Coker 2019) [24].

Term	Factor	Limits
Acidophile	pH	≥3
Alkaliphile	pH	≥9
Halophile	High salt concentration	1–4 M
Hyperthermophile and Thermophile	High temperatures	Hyperthermophile: above 80 °C (176 °F) Thermophile: between 45–122 °C (113–252 °F)
Piezophile (also called Barophile)	High pressures	~1100 bar
Psycrophile (also called Cryophile)	Low temperatures	≤−15 °C (5 °F)
Radiophile (also called Radioresistant)	UV radiation, cosmic rays, X-rays	1500 to 6000 Gy
Xerophile	Desiccating conditions	≤50% relative humidity
